# Supplementary material for: Alleviating Overgrazing Stress and Promoting Grassland Plant Regeneration via Root Exudate-Mediated Recruitment of Beneficial Bacteria
Source: Microorganisms. 2025 May 27;13(6):1225. doi: 10.3390/microorganisms13061225 (PMC12195006; doi:10.3390/microorganisms13061225)
Supplement: Supplementary file 1 [file microorganisms-13-01225-s001.zip › microorganisms-3598890-supplementary.pdf]

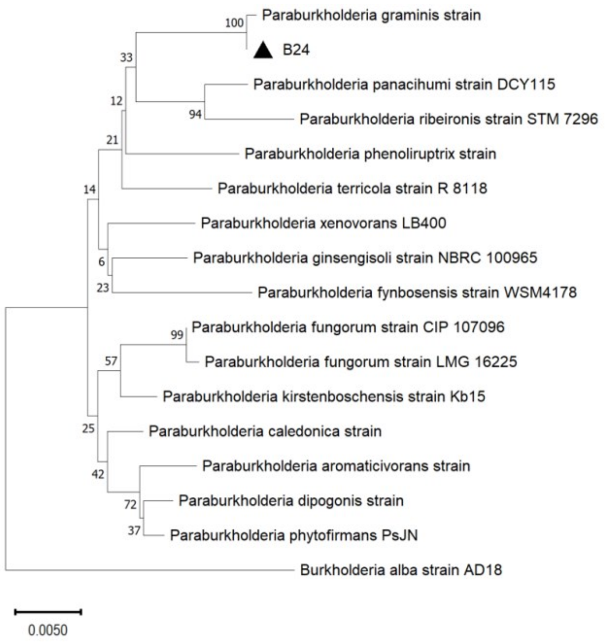

Figure S1. PGPR phylogenetic tree constructed based on 16S rDNA

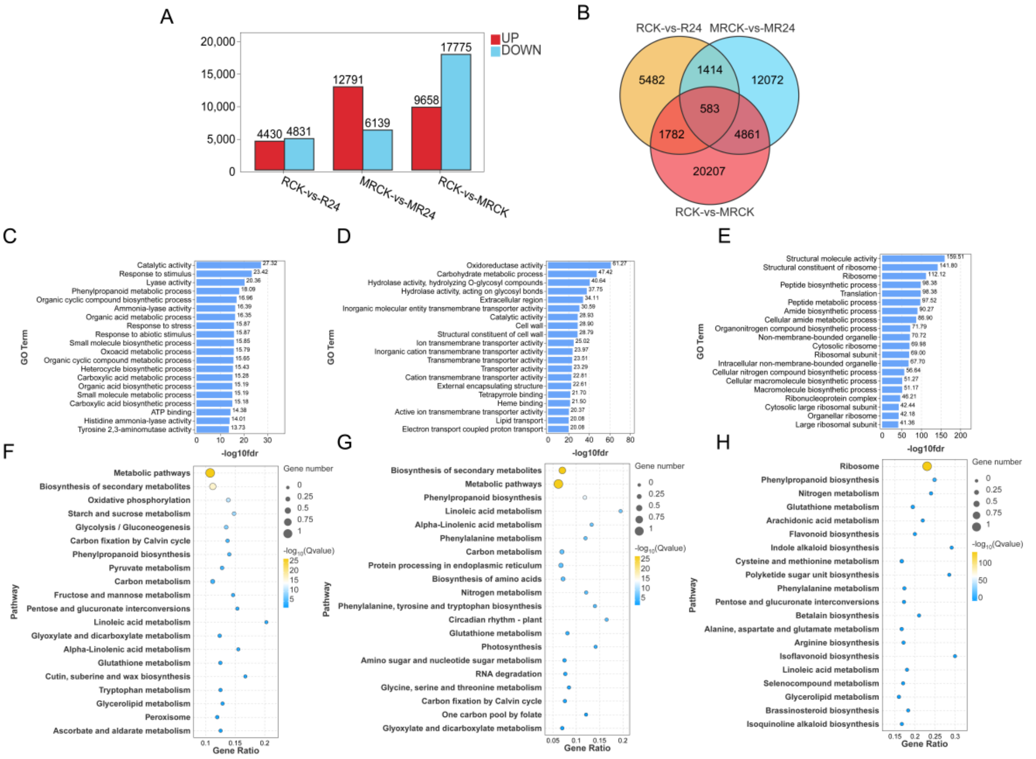

Figure S2. Number and function analysis of DEGs. (A)Histogram of DEGs; (B) Venn diagram of DEGs; (C-E) GO functional enrichment analysis of DEGs in RCK vs R24, MRCK vs MR24, and RCK vs MRCK, respectively; (F-H) KEGG functional enrichment analysis of DEGs in RCK vs R24, MRCK vs MR24, and RCK vs MRCK, respectively.

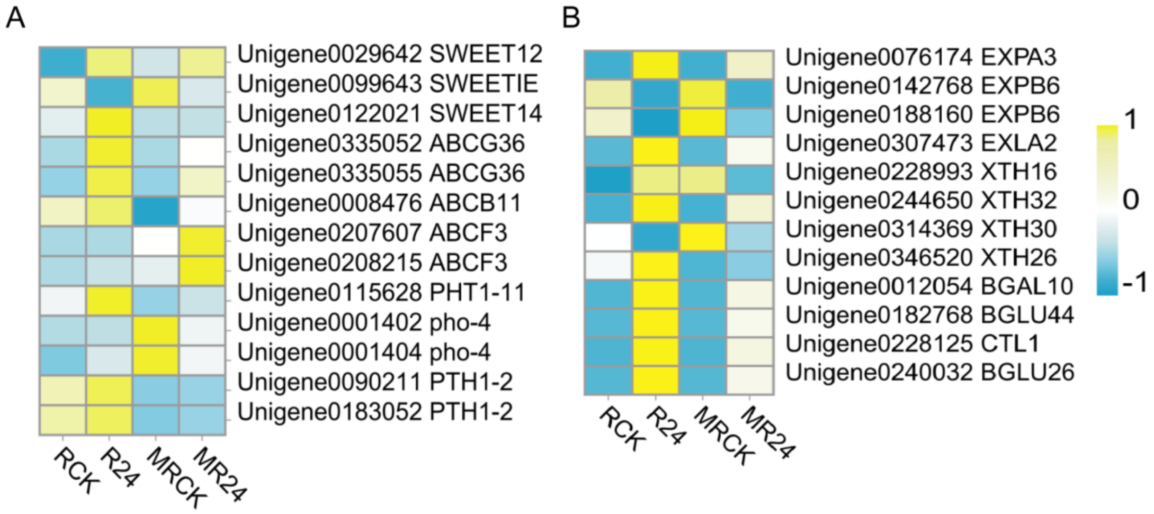

Figure S3. Expression of DEGs involved in plant growth and development. (A) DEGs involved in ion transport and sugar transport; (B) DEGs involved in cell wall elongation, expansion, and modification.

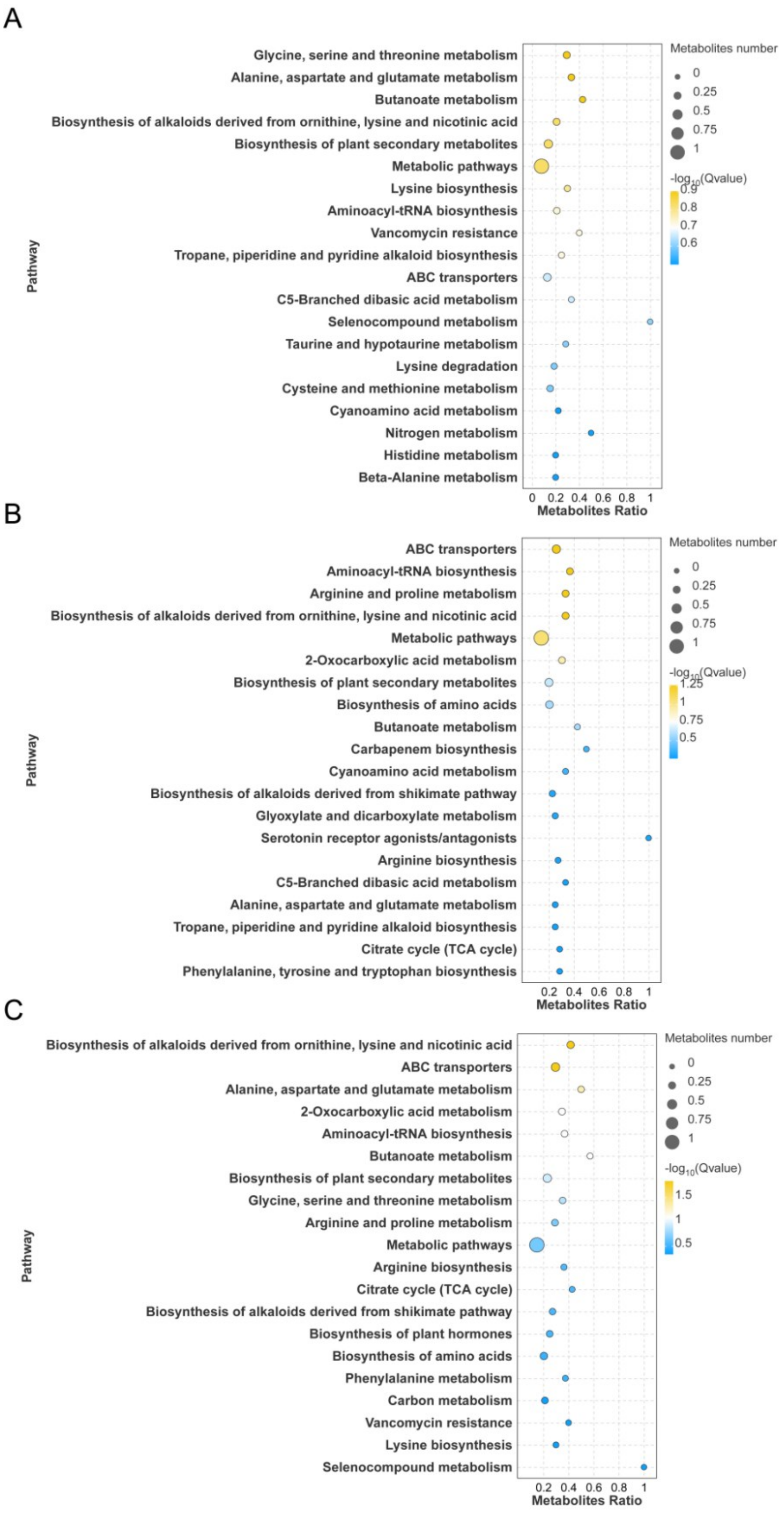

Figure S4. Bubble map of KEGG enrichment of differential metabolites (Top 20). (A) BCK vs B24; (B) MCK vs M24; (C) BCK vs MCK.

Table S1. Identification of dominant bacterial genera under overgrazing based on LEfSe analysis (LDA > 2, *P* < 0.05)

Feature	LDA score
Bacteria.Firmicutes.Bacilli.Bacillales.Bacillaceae.Bacillus	4.222194808
Bacteria.Proteobacteria.Alphaproteobacteria.Sphingomonadales.Sphingomonadaceae.Sphingopyxis	2.089293288
Bacteria.Actinobacteriota.Actinobacteria.Catenulisporales.Catenulisporaceae.Catenulispora	2.177127316
Bacteria.Acidobacteriota.Acidobacteriae.Acidobacteriales.Acidobacteriaceae_Subgroup_1.Granulicella	2.261618754
Bacteria.Proteobacteria.Alphaproteobacteria.Rhizobiales.Rhizobiaceae.Allorhizobium_Neorhizobium_Pararhizobium_Rhizobium	3.18025167
Bacteria.Firmicutes.Bacilli.Bacillales.Planococcaceae.Lysinibacillus	2.9345676
Bacteria.Actinobacteriota.Thermoleophilia.Gaiellales.Gaiellaceae.Gaiella	3.253157725
Bacteria.Bacteroidota.Bacteroidia.Chitinophagales.Chitinophagaceae.Pseudoflavitalea	2.153886579
Bacteria.Proteobacteria.Alphaproteobacteria.Rhizobiales.Rhizobiaceae.Mesorhizobium	2.691671384
Bacteria.Proteobacteria.Alphaproteobacteria.Azospirillales.Inquilinaceae.Inquilinus	2.305322851
Bacteria.Proteobacteria.Gammaproteobacteria.Burkholderiales.Nitrosomonadaceae.Nitrosospira	2.201887733
Bacteria.Actinobacteriota.Actinobacteria.Glycomycetales.Glycomycetaceae.Glycomyces	2.243141263
Bacteria.Bacteroidota.Bacteroidia.Chitinophagales.Chitinophagaceae.Niastella	2.668131261
Bacteria.Firmicutes.Bacilli.Paenibacillales.Paenibacillaceae.Paenibacillus	2.799054943
Bacteria.Actinobacteriota.Actinobacteria.Kineosporiales.Kineosporiaceae.Kineococcus	2.078408101
Bacteria.Proteobacteria.Alphaproteobacteria.Caedibacterales.Caedibacteraceae.Caedibacter	2.649897096
Bacteria.Firmicutes.Bacilli.Bacillales.Planococcaceae.Domibacillus	2.489808282
Bacteria.Firmicutes.Bacilli.Bacillales.Planococcaceae.Chungangia	2.025094619
Bacteria.Firmicutes.Bacilli.Bacillales.Bacillaceae.Oceanobacillus	2.574026004
Bacteria.Cyanobacteria.Cyanobacteriia.Cyanobacteriales.Coleofasciculaceae.Microcoleus_PCC_7113	3.162911701
Bacteria.Actinobacteriota.Thermoleophilia.Solirubrobacterales.Solirubrobacteraceae.Solirubrobacter	2.884118815
Bacteria.Actinobacteriota.Thermoleophilia.Solirubrobacterales.Solirubrobacteraceae.Conexibacter	2.571688538
Bacteria.Actinobacteriota.Actinobacteria.Bifidobacteriales.Bifidobacteriaceae.Bifidobacterium	2.0895116
Bacteria.Actinobacteriota.Actinobacteria.Streptomycetales.Streptomycetaceae.Streptomyces	3.137334367
Bacteria.Actinobacteriota.Actinobacteria.Frankiales.Frankiaceae.Jatrophihabitans	2.617863074

Bacteria.Actinobacteriota.Actinobacteria.Micromonosporales.Micromonosporaceae.Dactylosporangium	2.641553627
Bacteria.Actinobacteriota.Actinobacteria.Streptosporangiales.Thermomonosporaceae.Actinoallomurus	2.363554065
Bacteria.Actinobacteriota.Actinobacteria.Propionibacteriales.Nocardioidaceae.Kribbella	3.176028316
Bacteria.Proteobacteria.Gammaproteobacteria.Burkholderiales.Burkholderiaceae.Burkholderia_Caballeronia_Paraburkholderia	2.747977626
Bacteria.Firmicutes.Bacilli.Bacillales.Planococcaceae.Sporosarcina	2.655904317

Table S2. Growth promoting characterization of *Paraburkholderia graminis* (B24)

**Growth-promoting characterization**	**Means±SE**
Phosphate solubilization production (μg/mL)	179.90±2.59
IAA content (μg/mL)	25.33±2.17
ACC deaminase (IU/L)	178.12±3.48
Nitrogenase activity (IU/L)	199.13±0.74

Table S3. Quality statistics of RNA sequencing data

**Sample**	**RawDatas**	**CleanData(%)**	**Q20(%)**	**Q30(%)**	**GC(%)**
RCK-1	37978752	37805664 (99.54%)	5529010307 (97.67%)	5286614387 (93.38%)	3016745032 (53.29%)
RCK-2	37278080	37106470 (99.54%)	5416899912 (97.48%)	5170006987 (93.04%)	2961841210 (53.30%)
RCK-3	40881188	40671116 (99.49%)	5910373839 (97.03%)	5607881179 (92.07%)	3235145143 (53.11%)
R24-1	41565822	41436248 (99.69%)	6040068622 (97.84%)	5912891328 (95.78%)	3157472692 (51.15%)
R24-2	41795732	41662142 (99.68%)	6071134075 (97.85%)	5942274351 (95.78%)	3160674640 (50.94%)
R24-3	41219328	41086228 (99.68%)	5973320672 (97.91%)	5861455424 (96.08%)	3126778908 (51.25%)
MRCK-1	36692636	36548632 (99.61%)	5341343227 (97.59%)	5106905558 (93.31%)	2937081885 (53.66%)

MRCK-2	36424640	36310874 (99.69%)	5301984653 (97.50%)	5062527731 (93.09%)	2897550335 (53.28%)
MRCK-3	42413742	42217028 (99.54%)	6148927633 (97.26%)	5851729760 (92.56%)	3392112390 (53.65%)
MR24-1	39474226	39337606 (99.65%)	5728131912 (97.85%)	5584641551 (95.40%)	3122696671 (53.34%)
MR24-2	37290282	37179730 (99.70%)	5420108230 (98.09%)	5324425273 (96.36%)	2924136646 (52.92%)
MR24-3	39424612	39295792 (99.67%)	5724894046 (97.99%)	5617996511 (96.17%)	3124612474 (53.49%)

Table S4. Information on primers used in the qPCR assay

Genes	Primer name	Primer sequence（5’→3’）
Actin	F	ATTGTGCTCAGTGGTGGGTCA
	R	CCAATCCAAACACTGTACTTCCTC
NRT2.1	F	GATCTTGGTGCCCGCTACTT
	R	TGTAAGCCCGGAGATGATGC
GLN1-3	F	CGGGAATCAAGATCTCGGGG
	R	TCCGTCGAGAAGTTCGTGTG
ASP3	F	CCGTGTTCGCATCGCCT
	R	CGGTAAGCACCAACTCCCAA
panP	F	GGAGAATGTCGTGGTGCAGA
	R	TGCTCTTCCCCTTCCACCG
MDH1	F	AACACCAACGCACTGATCCT
	R	CAGCCGATGTCTGCACCTTA
SS2	F	GCTCGTGGACTTCTTTGGGA
	R	GTTGCAGCCATTTTGCGGTA
XTH26	F	CGCCCTGCATGATCGTCT
	R	TGCTCCTGACCGCCG

SWEET12	F	TGATCAGGACCAAGAGCGTG
	R	TGCTGAAGTGGCAGTTGGTT
PHT1-11	F	CTCACCCTCAAGGGCGAAGT
	R	CGACATCCTTGTCCACATGC
